# Assessment of Exercise Intolerance and Evaluation for HFpEF: A Literature Review of Pathophysiology, Diagnosis, and Management

**DOI:** 10.3390/pathophysiology33030063

**Published:** 2026-08-20

**Authors:** Ali Moradi, Kurt Ramey, Kutiba Tabbaa, Abdullah Sahyouni, Kevin Sanchez Garcez, Shivanshu Kumar, Elaine Pan, Grant Barton, Olugbenga Oyesanmi, Robert Subbiondo

**Affiliations:** 1 HCA Florida Blake Hospital, University of South Florida (USF) Morsani College of Medicine, Bradenton, FL 34209, USAolugbenga.oyesanmi@hcahealthcare.com (O.O.);; 2 Department of Medicine, USF Health Morsani College of Medicine, Tampa, FL 33602, USA; kumars1@usf.edu (S.K.);

**Keywords:** HF, HFpEF, heart failure, exercise intolerance

## Abstract

Heart failure with preserved ejection fraction (HFpEF) is a type of heart failure in which the ejection fraction remains within the normal range (≥50%); however, patients still experience typical symptoms of heart failure. One of the most common manifestations in this population is exercise intolerance, which in some cases may be the only presenting symptom. Exercise intolerance refers to a reduced capacity to perform physical activity. Several processes, including impaired cGMP–PKG signaling, increased collagen deposition, endothelial dysfunction, increased arterial stiffness, pulmonary hypertension, and vascular remodeling, contribute to the pathophysiology of HFpEF. The disruption of these fundamental physiological processes significantly impairs exercise capacity, leading to attenuated increases in heart rate, stroke volume, and/or contractility, along with abnormal ventricular–vascular coupling during exertion. When EI is suspected, a spectrum of diagnostic modalities—from simple, low-cost tools such as the 6 min walk test to advanced imaging—can be used to evaluate its presence and its association with HFpEF. When EI in HFpEF is diagnosed, management should focus on addressing this limitation to improve quality of life and reduce morbidity and mortality. Exercise training and pharmacological therapies, such as SGLT2 inhibitors, may be beneficial in this population. In conclusion, EI is a common manifestation of HFpEF that significantly impacts patients’ quality of life. Understanding its underlying mechanisms and addressing it appropriately are essential to improving patient outcomes.

## 1. Introduction

HFpEF is a clinical syndrome caused by impaired ventricular filling and diastolic dysfunction. It is characterized by a left ventricular ejection fraction (EF) of ≥50% in the presence of typical symptoms of heart failure [1]. The prevalence of HFpEF continues to increase each year, particularly among elderly patients. It is commonly associated with comorbid conditions such as uncontrolled hypertension, obesity, coronary artery disease (CAD), and chronic kidney disease (CKD) [2]. Patients with HFpEF can present with a wide range of symptoms, from nonspecific exertional dyspnea to more severe, typical heart failure manifestations, including peripheral edema, orthopnea, and signs of volume overload. One of the most common symptoms in HFpEF is exercise intolerance (EI), defined as a diminished capacity to engage in physical activity. EI may manifest as generalized weakness, fatigue, or exertional dyspnea and is associated with reduced quality of life and increased mortality [3,4]. Because these manifestations are nonspecific, HFpEF should be considered in patients with relevant risk factors and comorbidities. Initial evaluation may include clinical history, NYHA functional classification, validated heart failure questionnaires, and objective assessment of exercise capacity [4,5]. The mechanisms underlying EI are multifactorial and are discussed in detail below. EI may remain unrecognized due to its nonspecific presentation and is often underdiagnosed, particularly in elderly patients with multiple comorbidities. This review aims to provide a comprehensive and up-to-date overview of EI in patients with HFpEF, with a focus on current evidence and a structured approach to its pathophysiology, risk factors, and imaging modalities for assessment. In addition, we evaluate the impact of various types of exercise training and therapeutic interventions on EI. Our comprehensive review of the current literature aims to improve understanding, diagnosis, evaluation, and management of this condition, ultimately enhancing patients’ quality of life.

### 1.1. Definition and Classification of Heart Failure

The 2022 AHA/ACC/HFSA guideline defines heart failure (HF) as a clinical syndrome with symptoms and/or signs due to structural or functional cardiac abnormalities causing elevated filling pressures and/or reduced cardiac output, supported by natriuretic peptides or objective evidence of congestion. HF is stratified according to left ventricular ejection fraction (LVEF) estimations into HF with reduced EF (≤40%), HF with mildly reduced EF (41–49%), HF with preserved ejection fraction (≥50%), and HF with improved EF, with HFpEF requiring not only preserved EF but also evidence of elevated LV filling pressures and structural heart disease [6]. HFrEF is typically characterized by eccentric remodeling, chamber dilation, reduced end-systolic elastance (Ees), and impaired contractile reserve. In contrast, HFpEF more commonly exhibits concentric remodeling or hypertrophy, preserved or elevated Ees at rest, increased passive myocardial stiffness, and impaired relaxation [7,8]. These structural and functional distinctions contribute to the different clinical and hemodynamic manifestations of the two syndromes.

### 1.2. Pathophysiology of Heart Failure with Preserved Ejection Fraction (HFpEF)

One of the central mechanistic paradigms in HFpEF involves impaired nitric oxide (NO) bioavailability and downstream cyclic guanosine monophosphate (cGMP)-protein kinase G (PKG) signaling. Paulus et al. proposed that systemic inflammation associated with cardiometabolic comorbidities leads to coronary microvascular endothelial inflammation and oxidative stress, which reduces NO production and increases reactive oxygen species [9]. Impaired cGMP–PKG signaling also contributes to increased cardiomyocyte stiffness through altered titin phosphorylation. Titin is a giant sarcomeric protein that functions as a molecular spring and regulates passive cardiomyocyte tension and ventricular compliance. Reduced PKG-mediated titin phosphorylation increases titin-based passive stiffness and reduces left ventricular distensibility. Active myocardial relaxation also depends on efficient intracellular calcium handling. Following systole, calcium is removed from the cytosol primarily through sarco/endoplasmic reticulum calcium ATPase 2a (SERCA2a)-mediated reuptake into the sarcoplasmic reticulum, which is regulated by phospholamban, and through extrusion by the sodium–calcium exchanger (NCX). In some HFpEF phenotypes, impaired calcium reuptake or extrusion prolongs actin–myosin interaction and delays myocardial relaxation. In patients with impaired relaxation, more time is required for left ventricular pressure to decline sufficiently and for adequate ventricular filling to occur. During exercise or tachycardia, diastole shortens disproportionately as the heart rate increases, leaving insufficient time for complete myocardial relaxation and ventricular filling. This limitation reduces end-diastolic volume and stroke volume reserve while further increasing left ventricular filling pressures. The resulting elevation in pulmonary venous pressure and inadequate augmentation of cardiac output contribute to exertional dyspnea and exercise intolerance [8,9,10,11,12].

Interstitial fibrosis is a prominent feature of the myocardium in HFpEF. Collagen type I deposition, enhanced cross-linking through advanced glycation end products, and reduced matrix metalloproteinase activity contribute to increased extracellular stiffness within the myocardium [13]. Another important mechanism in HFpEF is coronary microvascular rarefaction and endothelial dysfunction, which reduce myocardial perfusion reserve [14]. Decreased capillary density and impaired vasodilatory response promote subendocardial ischemia, which may drive fibrosis and impaired relaxation. Microvascular inflammation represents a central link between systemic comorbidity and myocardial dysfunction.

Arterial stiffness has been known to increase with aging; when coupled with hypertension, it is exaggerated in HFpEF. Both increased pulse wave velocity and early wave reflection augment late systolic load, which subsequently impairs relaxation and increases myocardial oxygen demand [15]. The interaction between elevated arterial elastance (Ea) and preserved but stiff Ees leads to suboptimal mechanical efficiency. This decrease in mechanical efficiency is highlighted during exercise, where limited augmentation of Ees combined with elevated Ea results in inadequate stroke volume reserve. This results in dynamic mismatch, which is the main mechanism of exertional intolerance in patients with HFpEF [16]. Pulmonary hypertension (PH) is found to complicate approximately half of HFpEF cases. Hemodynamically, this translates to combined pre- and post-capillary PH, which is characterized by elevated pulmonary artery wedge pressure and increased pulmonary vascular resistance. The chronic elevation in left-sided filling pressures induces pulmonary vascular remodeling, including both medial hypertrophy and intimal fibrosis [17]. Over time, right ventricular (RV) afterload increases beyond adaptive capacity, which subsequently results in RV–pulmonary arterial uncoupling. RV–pulmonary arterial coupling can be quantified by the ratio of RV end-systolic elastance to arterial elastance. In HFpEF-associated PH, progressive uncoupling has been associated with adverse outcomes [18,19]. Table 1 provides a summary of the different pathophysiological mechanisms and their underlying processes in HFpEF.

### 1.3. Cardiovascular Response to Exercise in Healthy Individuals

In healthy individuals, exercise forces an immediate increase in metabolic demand that requires a rapid augmentation of oxygen delivery to organs and muscle tissue. Within seconds of exercise onset, reduction in parasympathetic response and sympathetic activation increases the heart rate and myocardial contractility, producing a near-linear rise in cardiac output (CO) with workload [20,21,22]. Concomitantly, the arterial baroreflex resets, which allows for higher operating pressures without reflex bradycardia, while the muscle metaboreflex responds to increasing metabolic products and central command further modulates chronotropy, inotropy, and systemic vascular resistance [20,21]. This prompts the redistribution of blood flow, which occurs through α-adrenergic-mediated vasoconstriction in both splanchnic and renal beds alongside metabolite-driven vasodilation in active skeletal muscle. This lowers the total peripheral resistance despite increasing the mean arterial pressure, These autonomic and vascular adjustments integrate to optimize ventriculoarterial coupling and preserve the perfusion pressure during progressive exercise [20,22].

In the heart, stroke volume (SV) increases during early and moderate exercise through enhanced venous return from the skeletal muscle and respiratory system, augmented diastolic suction, and increased contractility that is consistent with the Frank–Starling mechanism [20,22]. In most individuals, SV plateaus at approximately 40% to 60% of its maximal capacity, after which any further increase in CO is predominantly heart rate-mediated [20]. Exercise is also characterized by an approximate five-fold increase in coronary blood flow proportional to myocardial oxygen consumption, which is facilitated by metabolic vasodilation and endothelial nitric oxide signaling [23]. Simultaneously, large-artery compliance and wave reflection patterns dynamically adjust, influencing systolic load and pulse pressure during physical exertion [24]. These coordinated cardiac, autonomic, and vascular responses allow healthy individuals to increase oxygen delivery in proportion to metabolic demand during exercise [20,22,25].

### 1.4. Exercise Intolerance in Patients with HFpEF

Exercise intolerance in HFpEF refers to a reduced ability to perform physical activity and is commonly manifested by exertional dyspnea, fatigue, or both. It may be objectively quantified through reduced aerobic exercise capacity, including measurements such as peak oxygen consumption and 6 min walk distance [26]. Its clinical severity varies considerably among patients and may not correspond directly to abnormalities observed on resting cardiac evaluation. Because EI is nonspecific and may also result from pulmonary disease, anemia, obesity, deconditioning, or musculoskeletal limitations, further evaluation is required to determine whether the symptoms are attributable to HFpEF. The central cardiac, pulmonary vascular, peripheral vascular, and skeletal muscle mechanisms contributing to EI in HFpEF are discussed in the following section.

### 1.5. Pathophysiology of Exercise Intolerance in HFpEF

In HFpEF, EI is commonly interpreted as impaired reserves across multiple steps of the oxygen pathway, with cardiac constraints frequently including chronotropic incompetence (reduced heart rate reserve and/or abnormal recovery), impaired systolic/contractile and stroke volume reserve despite preserved resting EF, and abnormal ventricular–vascular interactions that can limit forward output during exertion. Invasive exercise studies also show exaggerated elevations in left-sided filling pressures with exercise (captured by peak exercise PCWP and/or PCWP/CO slope), and these exercise filling-pressure responses are associated with reduced peak VO2 in HFpEF cohorts [12]. Coronary microvascular dysfunction (CMD) has also been reported as prevalent in well-defined HFpEF cohorts, including a prospective multicenter study demonstrating a high prevalence of CMD in HFpEF [27]. In addition, in at least one cohort using Cardiac MRI and CPET, myocardial perfusion reserve was reduced in HFpEF and correlated positively with VO2max, supporting CMD as a potential contributor to exertional limitation and EI in subsets rather than a universal mechanism across all HFpEF phenotypes [28]. Vascular factors may further constrain exercise reserve. Arterial/aortic stiffening and increased wave reflections appear more evident during exercise than at rest in some studies and correlate with higher cardiac filling pressures and reduced cardiac output reserve [15].

Extracardiac contributors are also prominent and can be mechanistically linked to EI through both pulmonary and peripheral domains. Pulmonary studies describe reduced lung diffusing capacity during exercise in HFpEF, attributable in part to impaired alveolar–capillary membrane conductance (Dm) and limited augmentation of diffusing capacity with increasing work [29,30,31]. Invasive work also demonstrates abnormalities in pulmonary vascular reserve and impaired RV–pulmonary artery coupling during exercise in HFpEF, especially as filling pressures and pulmonary pressures rise [18,31].

**Figure 1:**
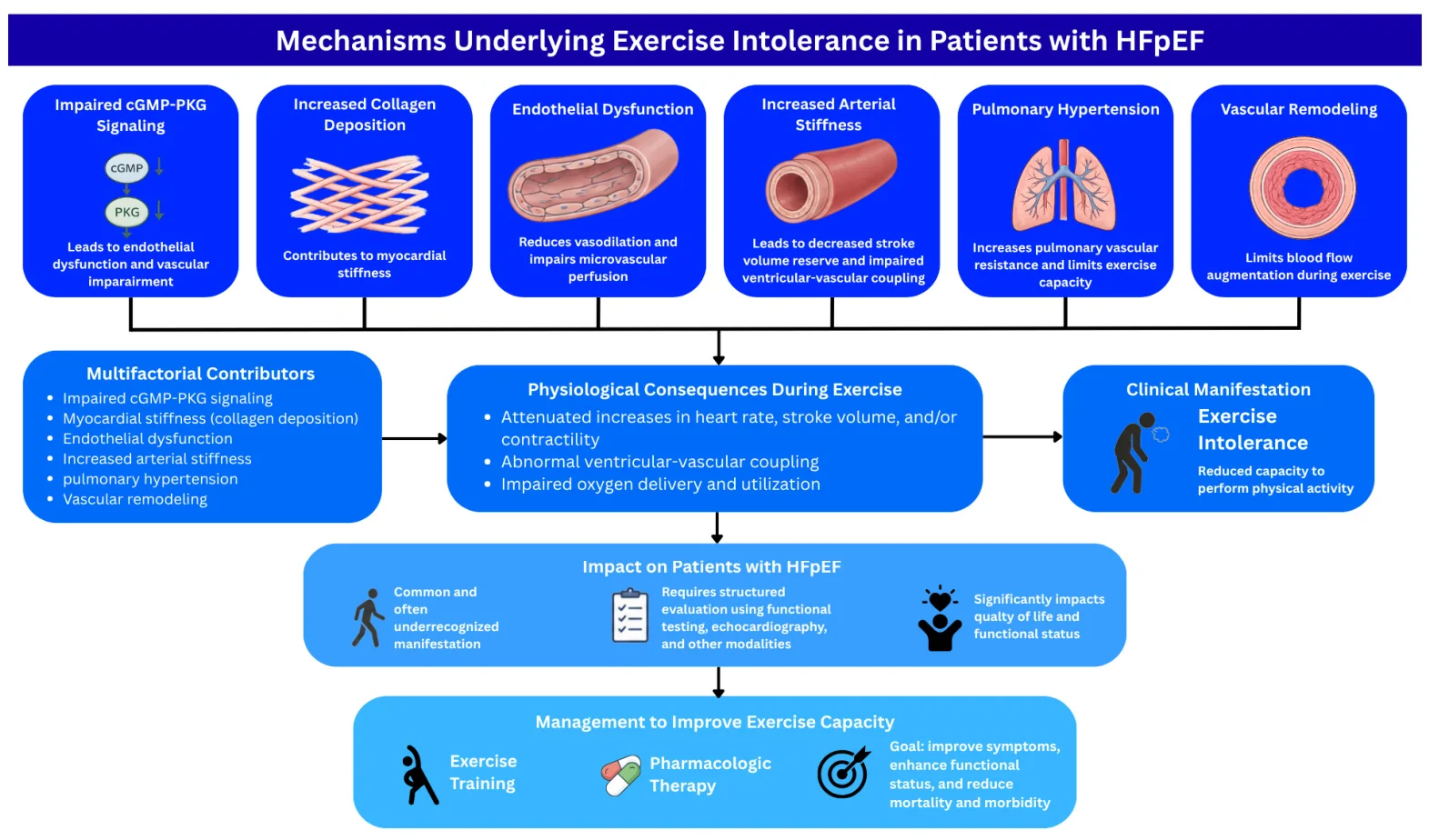
Mechanisms underlying exercise intolerance in patients with heart failure with preserved ejection fraction (HFpEF). Note: Arrows indicate the direction of mechanistic progression or downstream physiological consequences; ↓ indicates decreased or reduced activity/signaling.

Peripheral skeletal muscle dysfunction is also an important contributor to exercise intolerance in HFpEF. According to the Fick principle, oxygen consumption during exercise depends on both cardiac output and the peripheral extraction of oxygen from the blood. Therefore, even when systemic oxygen delivery is relatively preserved, impaired skeletal muscle oxygen extraction can substantially limit peak oxygen consumption. In a study that directly measured the arterial–mixed venous oxygen content difference during exercise, impaired peripheral oxygen extraction was a major determinant of reduced peak VO_2_ in HFpEF and represented the predominant exercise-limiting mechanism in a substantial subset of patients [32]. Structural abnormalities within skeletal muscle may contribute to this impaired oxygen utilization. Muscle biopsy studies in older patients with HFpEF have demonstrated a lower proportion of oxidative type I muscle fibers and a reduced capillary-to-fiber ratio, both of which were associated with lower peak VO_2_ [33,34]. Reduced capillary density limits oxygen diffusion from the circulation to active myocytes, while the shift away from oxidative fibers decreases the muscle’s capacity for sustained aerobic metabolism. In addition, reduced skeletal muscle mitochondrial content, oxidative capacity, and respiratory function have been reported in HFpEF and correlate with poorer exercise performance [34]. These abnormalities promote earlier reliance on anaerobic metabolism, accelerated metabolite accumulation, and premature skeletal muscle fatigue during physical activity. Thus, peripheral skeletal muscle dysfunction contributes to exercise intolerance through impaired oxygen delivery at the microvascular level, reduced oxygen extraction, and diminished mitochondrial utilization, independently of and in combination with central cardiac limitations. The major cardiac, pulmonary vascular, peripheral vascular, and skeletal muscle mechanisms contributing to exercise intolerance in HFpEF are summarized in Figure 1.

### 1.6. Comorbidities and Gender and Their Relationship with Exercise Intolerance in HFpEF

Obesity is common in HFpEF and appears to define a distinct exercise intolerance phenotype. Obokata et al. found that obese HFpEF patients (vs. nonobese HFpEF) had higher resting and exercised PCWP, greater exercise-induced pulmonary hypertension, and hemodynamic indices consistent with enhanced pericardial restraint [35]. A randomized trial by Kitzman et al. showed that among obese older patients with HFpEF, caloric restriction or aerobic exercise increased peak VO2, with possible additive effects [36]. Sex-related physiologic differences in HFpEF have been described in invasive exercise-phenotyping cohorts. In a physiologically defined HFpEF cohort undergoing invasive CPET, Lau et al. reported that women (vs. men) had lower exercise peripheral O2 extraction, lower LV and RV systolic reserve, and a steeper PCWP/CO slope, consistent with greater impairment in diastolic reserve, as revealed during exercise [37]. In an independent cohort using complementary invasive hemodynamics and echocardiography, Beale et al. found that women had higher PCWP indexed to peak exercise workload and lower systemic and pulmonary arterial compliance during exercise, alongside evidence of more abnormal peripheral oxygen kinetics (e.g., greater lactate rise per workload) [38].

### 1.7. Different Modalities to Assess HFpEF in Patients with Nonspecific Exercise Intolerance

Patients presenting with nonspecific EI and suspected HFpEF require a structured stepwise diagnostic approach, as the diagnosis often cannot be established by any single clinical or imaging data point alone [39,40]. Contemporary evaluation relies on complementary modalities that help define the likelihood of HFpEF and identify objective evidence of elevated filling pressures, including 6MWT, resting and exercise cardiography, CPET, and, when uncertainty persists, invasive exercise hemodynamic testing such as exercise right heart catheterization or invasive CPET [39,40,41,42]. These approaches are all particularly important because many patients with HFpEF have normal resting findings and develop abnormal hemodynamics only during physiological stress [12,41]. Accordingly, the diagnostic workup is best performed in a staged fashion, using noninvasive assessment to guide escalation to more definitive invasive testing when diagnosis remains uncertain (Figure 1) [39,40].

### 1.8. Practical Diagnostic Algorithm for Exercise Intolerance and Suspected HFpEF

Evaluation should begin by confirming the presence and severity of exercise intolerance through the clinical history and, when appropriate, objective functional assessment with the 6 min walk test [43,44]. The initial evaluation should include physical examination, electrocardiography, natriuretic peptide measurement, routine laboratory testing, and resting transthoracic echocardiography [39,40]. Resting echocardiography is used to identify preserved ejection fraction, structural heart disease, diastolic dysfunction, and evidence of elevated left ventricular filling pressures [45,46]. At this stage, clinicians should also assess for alternative or contributing causes of exertional dyspnea and fatigue, including pulmonary disease, anemia, myocardial ischemia, valvular heart disease, arrhythmias, obesity, physical deconditioning, medication effects, and musculoskeletal or neuromuscular limitations [40,42].

The clinical likelihood of HFpEF should subsequently be estimated using a validated framework such as the H_2_FPEF or HFA-PEFF score. A high probability, together with objective structural or functional evidence of elevated left ventricular filling pressures, supports the diagnosis of HFpEF. A low probability should prompt investigation of alternative causes of exercise intolerance, although additional testing may remain appropriate when clinical suspicion is discordantly high [39,40]. Patients with an intermediate probability or inconclusive resting findings should undergo functional evaluation with exercise echocardiography and/or cardiopulmonary exercise testing [39,41,42]. The 6 min walk test can document the presence and severity of functional limitation but cannot independently distinguish HFpEF from pulmonary, peripheral, or deconditioning-related causes [43,44].

Exercise echocardiography may support the diagnosis of HFpEF by demonstrating an abnormal increase in left ventricular filling pressure during exertion, particularly in patients with normal or indeterminate resting findings [41,46]. Noninvasive cardiopulmonary exercise testing can quantify the severity of exercise impairment and help identify cardiac, pulmonary, peripheral, or mixed patterns of physiological limitation [42]. When exercise echocardiographic findings indicate elevated filling pressures and are consistent with the clinical presentation, HFpEF is strongly supported [39,41,46]. When noninvasive exercise testing remains inconclusive or discordant despite persistent clinical suspicion, exercise right heart catheterization or invasive cardiopulmonary exercise testing should be performed to directly measure filling pressures, pulmonary pressures, and cardiac output during physiological stress [12,41,42,46].

**Figure 2:**
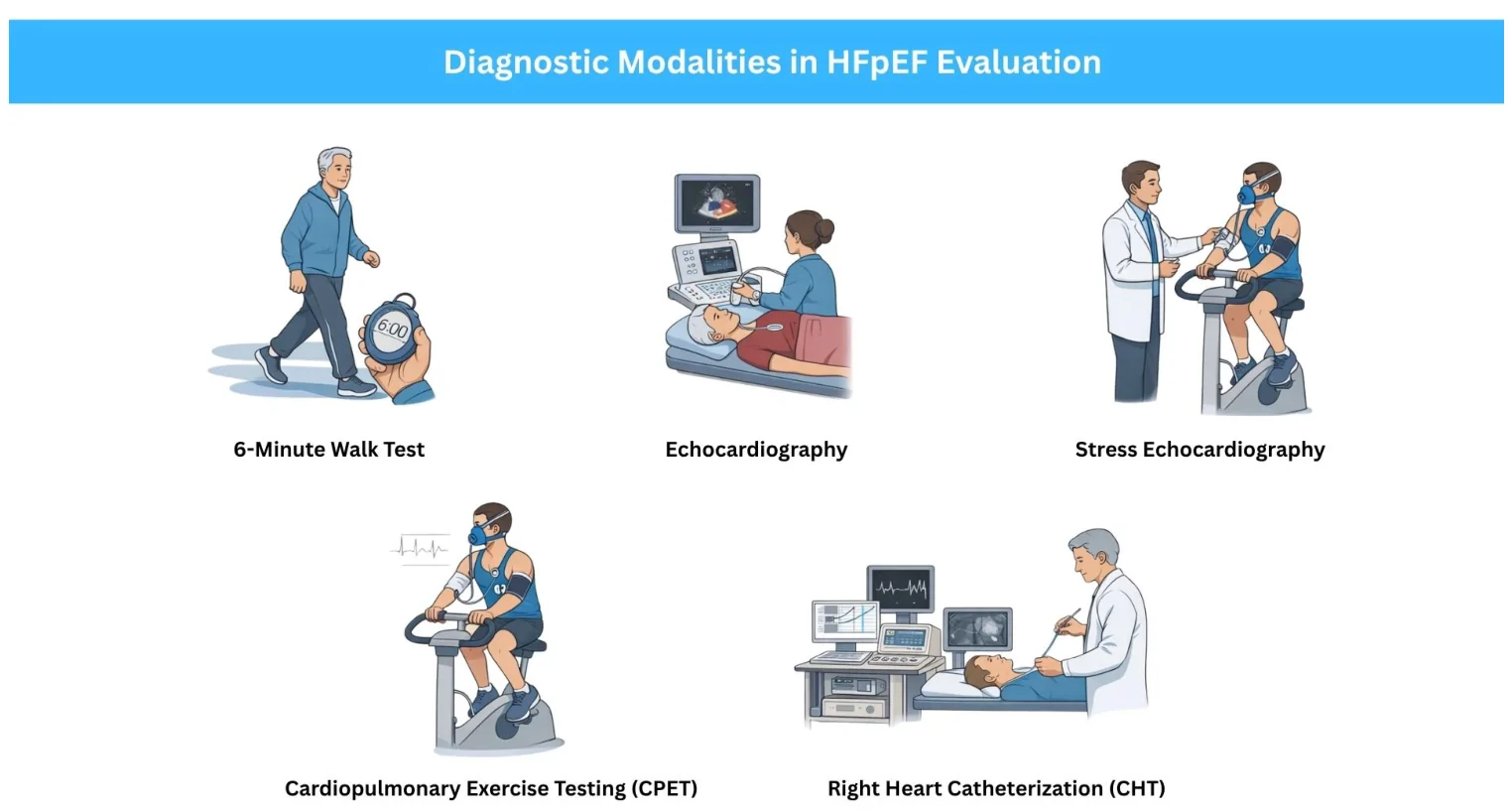
Various modalities used in the evaluation and diagnosis of heart failure with preserved ejection fraction (HFpEF).

An abnormal exercise-induced elevation in pulmonary capillary wedge pressure supports the presence of HFpEF physiology, including in patients whose resting filling pressures are normal [12,41]. Conversely, normal cardiac filling pressures during exercise make HFpEF less likely and should prompt a more focused evaluation for alternative cardiac, pulmonary, vascular, hematologic, metabolic, skeletal muscle, or deconditioning-related causes of exercise intolerance [41,42]. The principal diagnostic modalities used to evaluate exercise intolerance and support the diagnosis of HFpEF are summarized in Figure 2.

### 1.9. Six-Minute Walk Test in Patients with Nonspecific Exercise Intolerance

The cheapest and easiest way to assess exercise and functional capacity is the 6 min walk test (6MWT). During this test, the patient walks for six minutes at their own pace, and the total distance walked is recorded. This test provides a nonspecific assessment of cardiovascular, pulmonary, and skeletal muscle function. In patients with suspected HFpEF, the 6MWT can provide an initial objective assessment of exercise intolerance as part of a broader diagnostic evaluation that includes the clinical history, physical examination, natriuretic peptide levels, resting echocardiography, and validated diagnostic scores such as H_2_FPEF and HFA-PEFF. A reduced walking distance supports the presence of clinically meaningful functional impairment and may indicate the need for further evaluation with exercise echocardiography, cardiopulmonary exercise testing, or invasive exercise hemodynamic testing when resting findings are inconclusive. However, the 6MWT cannot independently establish a diagnosis of HFpEF because performance may also be affected by age, body size, effort, obesity, pulmonary disease, anemia, musculoskeletal limitations, and deconditioning. Similarly, a preserved walking distance does not exclude early HFpEF, particularly in patients who develop abnormal elevations in cardiac filling pressures only during greater physiological stress [39,40,41,42].

This test is useful for staging disease severity and can also be used during hospitalization to evaluate treatment efficacy and predict mortality [4,43]. In patients with HFpEF, it provides valuable information regarding disease severity and prognosis. A walking distance of less than 300 m has been associated with decreased survival among patients with mild to moderate heart failure symptoms, such as fatigue and exertional dyspnea. Cavero-Redondo et al. compared the 6MWT with CPET for identifying reduced functional capacity, defined as a VO_2_max below 14 mL/kg/min, and reported a sensitivity of 78% and specificity of 79%. These values reflect the test’s ability to identify reduced functional capacity rather than its ability to diagnose HFpEF itself. While the 6MWT is a practical screening tool for functional capacity, its moderate accuracy may lead to false results, and CPET remains necessary when significant impairment is suspected [44].

### 1.10. Exercise Echocardiography in Patients with Nonspecific Exercise Intolerance

Echocardiography is a key tool in the diagnosis of HFpEF; however, unlike HFrEF, where a reduced ejection fraction makes the diagnosis more straightforward, HFpEF is more challenging to identify. In HFpEF, echocardiographic evaluation primarily focuses on detecting diastolic dysfunction and estimating elevated left atrial filling pressure. Parameters that provide diagnostic clues include the transmitral flow pattern, early diastolic mitral annular tissue velocity (e′), the ratio of early mitral inflow velocity to e′ velocity (E/e′ ratio), tricuspid regurgitation velocity (TRV), and pulmonary venous flow patterns. These parameters are highly specific for detecting elevated left atrial pressure but have limited sensitivity [45,47]. According to the American Society of Echocardiography, the assessment of left atrial pressure should rely on a combination of parameters to compensate for the limited sensitivity of individual left ventricular diastolic function indices; however, even the multiparametric approach has been shown to have relatively low sensitivity. As a result, many patients with HFpEF may go unrecognized [45,48]. Resting and exercise echocardiography have complementary diagnostic roles in suspected HFpEF. Resting echocardiography evaluates preserved left ventricular ejection fraction, impaired myocardial relaxation, elevated left-sided filling pressures, and structural changes associated with chronically increased filling pressures. Exercise echocardiography is primarily used when resting findings are normal or indeterminate despite persistent exertional symptoms, as it may reveal abnormal elevations in filling pressure that occur only during physiological stress. The principal resting and exercise echocardiographic findings and their diagnostic roles are summarized in Table 2.

Therefore, in suspected HFpEF, pre-test probability using the H2FPEF and HFA-PEFF scores guides further evaluation. Patients with an intermediate probability may benefit from exercise stress echocardiography, whereas those with clearly low or high pre-test probability generally do not require stress testing [39,40,49]. Exercise stress testing can provide important information about the cardiovascular response to exertion, including changes in preload, systolic and diastolic function, chronotropic response, and afterload [50,51]. For HFpEF assessment, ASE favors supine bicycle exercise over treadmill testing because it enables real-time image acquisition throughout the study and offers greater safety, especially in older individuals [50,52]. For the assessment of unexplained dyspnea and suspected HFpEF, exercise echocardiography should evaluate TMF patterns, mitral annular e′ velocity, and TRV. The exercise E/e′ ratio serves as a surrogate for LV filling pressure, while exercise TRV provides complementary evidence of pulmonary pressure elevation. According to the updated ASE recommendations, diastolic exercise echocardiography is considered positive when the exercise average E/e′ ratio is ≥14 or the septal E/e′ ratio is ≥15, together with an exercise peak TR velocity ≥3.2 m/s. These findings support an abnormal exercise-induced elevation in LV filling pressure. Because Doppler measurements may be unobtainable or discordant during exercise, invasive exercise right heart catheterization may be warranted when exercise echocardiography is inconclusive [46,50].

### 1.11. Noninvasive CPET in Patients with Suspected HFpEF and Exercise Intolerance

Noninvasive CPET is used to quantify the severity of exercise intolerance and characterize the physiological pattern of limitation through measurements such as peak VO_2_ and ventilatory efficiency. In a Mayo Clinic study comparing patients with invasively verified HFpEF with those who had noncardiac dyspnea, an upright peak VO_2_ below 14 mL/kg/min had high specificity but limited sensitivity for HFpEF, whereas a peak VO_2_ above 20 mL/kg/min was sensitive for excluding HFpEF but had limited specificity. Intermediate values demonstrate substantial overlap and cannot independently establish or exclude the diagnosis. Noninvasive CPET is therefore most useful for documenting exercise impairment, identifying cardiac, pulmonary, peripheral, or mixed patterns of limitation, and determining whether additional exercise hemodynamic testing is warranted [42]. The complementary roles, strengths, and limitations of noninvasive CPET, exercise right heart catheterization, and invasive CPET in the evaluation of HFpEF are summarized in Table 3.

### 1.12. Invasive Exercise Hemodynamic Testing: Exercise Right Heart Catheterization and Invasive CPET

Invasive exercise hemodynamic testing is used when resting and noninvasive exercise evaluations remain inconclusive despite persistent suspicion for HFpEF. Exercise right heart catheterization directly measures pulmonary capillary wedge pressure, pulmonary artery pressures, and cardiac output during physiological stress and can reveal abnormal elevations in left-sided filling pressures that are absent at rest [12,41,46]. Invasive CPET combines these hemodynamic measurements with simultaneous gas exchange assessment, allowing a more comprehensive distinction among impaired cardiac output reserve, pulmonary vascular limitation, and abnormal peripheral oxygen extraction [42]. An abnormal exercise-induced elevation in PCWP supports HFpEF physiology, whereas normal exercise filling pressures make HFpEF less likely and should prompt evaluation for alternative causes of exercise intolerance [12,41]. Thus, exercise RHC provides definitive hemodynamic assessment, while invasive CPET provides additional mechanistic characterization when the cause of exercise limitation remains uncertain.

### 1.13. Exercise Training in Patients with Exercise Intolerance

Exercise training has consistently demonstrated improvements in functional capacity in patients with HFpEF across multiple randomized controlled trials, despite heterogeneity in training modalities. Both aerobic-based programs and high-intensity interval training (HIIT) interventions have been associated with significant increases in peak VO2, a central determinant of exercise tolerance. Trials, such as the ones listed in Table 4, uniformly report improvements in peak VO2, as well as gains in submaximal exercise performance, including 6 min walk distance and ventilatory efficiency. Additionally, quality of life metrics also demonstrate consistent improvement following structured interventions. Importantly, these benefits are observed across a range of program designs, including supervised aerobic training, combined aerobic-resistance regimens, and HIIT protocols, suggesting that the physiological response to exercise in HFpEF is robust and not restricted to a single training modality.

The improvement in exercise tolerance following structured exercise training appears to result predominantly from peripheral and integrative physiological adaptations rather than major changes in resting cardiac structure. Repeated aerobic exercise may increase skeletal muscle capillary density, mitochondrial content, oxidative enzyme activity, and the capacity for peripheral oxygen extraction, thereby increasing the arterial–venous oxygen difference during exertion. Exercise training may also improve endothelial nitric oxide-mediated vasodilation and the distribution of blood flow to active skeletal muscle while reducing arterial stiffness and systemic vascular resistance. These vascular adaptations can decrease afterload and improve ventricular–arterial coupling during exercise. In addition, improved autonomic balance, chronotropic response, and plasma volume expansion may enhance cardiac output and oxygen delivery during exertion. Collectively, these adaptations delay the transition to anaerobic metabolism, reduce premature skeletal muscle fatigue and ventilatory demand, and improve peak VO_2_ and submaximal exercise capacity. Mechanistic studies suggest that the increase in peak VO_2_ following endurance training in HFpEF is often driven largely by improved peripheral oxygen extraction, which helps explain why functional capacity may improve even when resting echocardiographic measures of diastolic function remain unchanged [53,54,55,56].

On the other hand, the effects of exercise training on cardiac structure and diastolic function remain inconsistent. As seen in Table 4, most trials report no significant changes in key echocardiographic parameters such as E/e′, left atrial volume index (LAVI), or left ventricular structural indices following intervention. However, Edelmann et al. represents a notable exception with improvements in E/e’ and indices of diastolic function; however, these findings have not been consistently replicated in subsequent studies [57]. Collectively, these findings across studies demonstrate that exercise training confers meaningful clinical benefits that may or may not be replicated in changes in traditional echocardiographic markers.

**Table 4:** Summary of Randomized Controlled Trials of Exercise Training in Patients with HFpEF.

First Author	Number of Participants (N)	Echocardiographic Findings	Type of EXERCISE	Outcomes Measured
**Mueller et al. [58]**	180	Diastolic parameters: E/e’ medial, e’ medial, LAVI—found no significant change compared to control	Aerobic Training: HIIT vs. moderate continuous vs. guideline-based activity	Primary: Peak VO2 Secondary: VE/VCO2 slope, workload at VT1, NT-proBNP, Quality of Life
**Angadi et al. [59]**	19	Diastolic dysfunction grade ↓, E velocity ↓, DT ↑ with HIIT; no change in LAVI, E/A, e′, E/e′, IVRT, EF; no changes in MICT	Aerobic Training: HIIT vs. MCT	Primary: Peak VO2 Secondary: VO2 at ventilatory threshold, VE/VCO2 slope, peak HR, peak RER, endothelial function, arterial stiffness
**Edelmann et al. [57]**	64	E/e′ ↓ (improved filling pressures), improved diastolic function indices; no major structural remodeling (LAVI unchanged)	Aerobic + resistance training	Primary: peak VO_2_ Secondary: 6MWT, Quality of Life (MLHFQ), ventilatory efficiency (VE/VCO_2_ slope), workload/exercise time
**Kitzman et al. 2016 [36]**	100	No significant change in diastolic function (E/e′ unchanged), no structural remodeling (LAVI unchanged)	Aerobic training (±caloric restriction; factorial design)	Primary: peak VO_2_ Secondary: 6MWT, Quality of Life (MLHFQ), body weight/composition, exercise time/workload
**Kitzman et al. 2010 [60]**	53 randomized (43 completed)	No significant changes in LV structure or diastolic function (E/A, DT, LV mass, EF all unchanged)	Aerobic training (supervised, moderate intensity)	Primary: peak VO_2_; Secondary: 6MWT, ventilatory anaerobic threshold (VAT), peak workload, exercise time, heart rate reserve, oxygen pulse, VE/VCO_2_ slope, Quality of Life (MLHFQ physical score)

Note: ↑ indicates increased or elevated; ↓ indicates decreased or reduced.

### 1.14. Effect of Current Treatment of HFpEF for Exercise Intolerance

Current therapeutic strategies for HFpEF have demonstrated variable and often modest effects on exercise intolerance, reflecting the syndrome’s multifactorial pathophysiology. Contemporary recommendations emphasize a phenotype- and comorbidity-directed approach to HFpEF management. The 2023 ACC Expert Consensus Decision Pathway prioritizes the identification and treatment of major contributors, including hypertension, obesity, diabetes, atrial fibrillation, coronary artery disease, chronic kidney disease, and obstructive sleep apnea, together with appropriate symptom control and disease-modifying therapy. The 2023 ESC Focused Update further recommends dapagliflozin or empagliflozin in patients with symptomatic HFpEF to reduce the risk of heart failure hospitalization or cardiovascular death [61,62]. Pharmacologic therapies that have reshaped HFpEF management, particularly sodium–glucose cotransporter 2 (SGLT2) inhibitors such as empagliflozin and dapagliflozin, have consistently reduced heart failure hospitalizations and improved clinical outcomes across large randomized trials such as EMPEROR-Preserved and DELIVER [63,64]. However, their direct impact on exercise capacity has been less pronounced. Although SGLT2 inhibitors may improve HF-related health status, their effects on objective exercise performance have been inconsistent across trials [65,66]. This disconnect highlights that while therapies can improve congestion, clinical outcomes do not necessarily normalize the physiological impairments that come with HFpEF. Structured exercise training remains an important nonpharmacologic component of HFpEF management; its effects on functional capacity and the physiological mechanisms underlying these benefits are discussed in the preceding section [53,56,67].

More recently, phenotype-targeted therapies have demonstrated meaningful improvements in exercise intolerance. The STEP-HFpEF trial showed that treatment with semaglutide, a GLP-1 receptor agonist, led to significant improvements in symptoms, physical limitations, and 6 min walk distance, alongside substantial weight loss [68]. The SUMMIT trial subsequently demonstrated that tirzepatide reduced the risk of the composite of cardiovascular death or worsening heart failure and improved health status and exercise capacity in patients with HFpEF and obesity [69]. These findings support obesity and cardiometabolic dysfunction as modifiable drivers of HFpEF rather than merely coexisting conditions. More broadly, the heterogeneous nature of HFpEF supports matching treatment to dominant clinical phenotypes and comorbidities, including obesity and metabolic dysfunction, hypertension, atrial fibrillation, chronic kidney disease, and pulmonary vascular disease [70]. Overall, these results suggest that targeting metabolic and inflammatory drivers of HFpEF can improve functional capacity by reducing cardiac loading conditions and enhancing peripheral efficiency. However, despite these advances, to date no single therapy has been able to fully restore exercise capacity within HFpEF. Instead, the current evidence supports a multimodal, phenotype-specific approach that integrates pharmacological therapy, structured training, and comorbidity management to address the mechanisms underlying exercise intolerance.

## 2. Limitations and Future Directions

This study provides a comprehensive overview of the pathophysiology of HFpEF and associated exercise intolerance; however, it was not conducted systematically, and relevant studies may have been missed. Additionally, data heterogeneity limits definitive conclusions. Despite this, our findings emphasize the importance of recognizing HFpEF in patients with nonspecific symptoms, even when echocardiography appears normal due to preserved ejection fraction. Future research should focus on systematic investigations and effective treatment strategies to improve outcomes in this population.

## 3. Conclusions

EI is an early and common feature of HFpEF that is often underrecognized, as resting echocardiography may be normal. It is multifactorial, involving impaired myocardial relaxation, vascular dysfunction, and abnormal cardiopulmonary coupling. When suspected, diagnostic tools such as exercise echocardiography, CPET, and right heart catheterization can help confirm the diagnosis. Management with exercise training and targeted medical therapy may improve symptoms and outcomes.

## Figures and Tables

**Table 1:** Overview of the Pathophysiology of Heart Failure with Preserved Ejection Fraction.

Pathophysiology in HFpEF	Mechanisms and Underlying Processes
Molecular and Cellular Mechanisms of HFpEF	↓ Nitric oxide bioavailability from microvascular inflammation and oxidative stress → impaired cGMP–PKG signaling
Titin-Mediated Cardiomyocyte Stiffness	Reduced PKG-mediated titin phosphorylation and altered titin properties → ↑ passive cardiomyocyte tension, ↑ myocardial stiffness, and ↓ ventricular compliance
Calcium Handling and Active Relaxation	Impaired SERCA2a-mediated Ca^2+^ reuptake, phospholamban regulation, and/or NCX-mediated Ca^2+^ extrusion → prolonged cytosolic Ca^2+^ and delayed myocardial relaxation; exercise-induced shortening of diastole leaves insufficient time for ventricular filling → ↓ end-diastolic volume, ↓ stroke volume reserve, and exercise intolerance
Extracellular Matrix Remodeling and Fibrosis	Collagen deposition, AGE cross-linking, and ↓ matrix metalloproteinase activity → ↑ myocardial stiffness and impaired relaxation
Coronary Microvascular Dysfunction (CMD)	Microvascular rarefaction and endothelial dysfunction → ↓ perfusion reserve and subendocardial ischemia
Ventricular–Arterial Coupling and Hemodynamic Load	↑ Arterial stiffness (↑ Ea) + stiff LV (Ees) → impaired coupling, ↓ stroke volume reserve, exertional intolerance
Right Ventricular–Pulmonary Vascular Coupling	↑ Left-sided filling pressures lead to pulmonary hypertension and vascular remodeling → right ventricular–pulmonary arterial uncoupling and adverse outcomes

Note: ↑ indicates increased or elevated; ↓ indicates decreased or reduced; → indicates leads to, results in, or contributes to.

**Table 2:** Resting and Exercise Echocardiographic Parameters Used in the Diagnostic Evaluation of HFpEF.

Assessment	Principal Echocardiographic Findings	Diagnostic Role
**Resting echocardiography**	Preserved LVEF ≥ 50%; transmitral E/A pattern; reduced mitral annular e′ velocity; increased septal E/e′ ≥ 15, lateral E/e′ ≥ 13, or average E/e′ ≥ 14; TR velocity ≥ 2.8 m/s or PASP ≥ 35 mmHg; supportive findings include LAVI > 34 mL/m^2^, left atrial reservoir strain ≤ 18%, pulmonary vein S/D ratio ≤ 0.67, and IVRT ≤ 70 ms	Confirms preserved EF and identifies resting diastolic dysfunction, elevated left atrial pressure, and structural or functional evidence supporting HFpEF. Elevated filling pressure at rest in an appropriate symptomatic patient supports the diagnosis.
**Exercise echocardiography**	Exercise average E/e′ ≥ 14 or septal E/e′ ≥ 15 together with peak TR velocity ≥ 3.2 m/s	Unmasks exercise-induced elevation in LV filling pressure when resting findings are normal or indeterminate. A positive study supports HFpEF; an inconclusive study may require invasive exercise hemodynamic testing.

**Table 3:** Comparison of Invasive and Noninvasive Cardiopulmonary Exercise Testing (CPET).

Assessment Domain	Noninvasive CPET	Exercise Right Heart Catheterization	Invasive CPET
**Primary purpose**	Quantifies exercise intolerance and characterizes the general physiological pattern of limitation	Directly evaluates cardiac and pulmonary hemodynamics during exercise	Integrates direct exercise hemodynamics with simultaneous gas exchange measurements
**Hemodynamic assessment**	No direct measurement of intracardiac pressures	Direct measurement of PCWP, pulmonary artery pressure, right atrial pressure, and cardiac output at rest and during exercise	Direct measurement of PCWP, pulmonary artery pressure, right atrial pressure, and cardiac output at rest and during exercise
**Gas exchange assessment**	Measures peak VO_2_, ventilatory efficiency, respiratory exchange ratio, and related exercise variables	Does not routinely provide comprehensive breath-by-breath gas exchange assessment	Measures peak VO_2_, ventilatory efficiency, respiratory exchange ratio, and arterial–venous oxygen content difference alongside hemodynamics
**Key HFpEF findings**	Reduced peak VO_2_ and abnormal ventilatory patterns may increase or decrease the likelihood of HFpEF but are not independently diagnostic	Exercise-induced elevation in PCWP supports HFpEF physiology, including when resting filling pressures are normal	Demonstrates elevated exercise filling pressures while also identifying impaired cardiac output reserve, pulmonary vascular limitation, or abnormal peripheral oxygen extraction
**Diagnostic role**	Documents functional impairment, identifies cardiac, pulmonary, peripheral, or mixed limitation patterns, and determines whether invasive testing is needed	Provides definitive assessment of whether abnormal left-sided filling pressures develop during exercise	Confirms HFpEF hemodynamics and provides additional mechanistic characterization of the cause of exercise intolerance
**Strengths**	Noninvasive, widely available, and useful for quantifying functional limitation	Direct and definitive measurement of exercise filling pressures	Most comprehensive physiological evaluation of unexplained exercise intolerance
**Limitations**	Cannot directly measure filling pressures or independently confirm HFpEF	Invasive and less informative regarding peripheral oxygen utilization without simultaneous gas exchange analysis	Invasive, resource-intensive, and available primarily at specialized centers

## Data Availability

Data is available on request due to privacy/ethical restrictions.
